# MicroRNA-148a inhibits migration and invasion of ovarian cancer cells via targeting sphingosine-1-phosphate receptor 1

**DOI:** 10.3892/mmr.2021.12298

**Published:** 2021-07-15

**Authors:** Zhengfang Wen, Shuzhen Zhao, Shanshan Liu, Ying Liu, Xiaorui Li, Shaoru Li

Mol Med Rep 12: 3775-3780, 2015; DOI: 10.3892/mmr.2015.3827

Following the publication of this paper, it was drawn to the Editors’ attention by a concerned reader that certain of the western blotting data shown in Fig. 4A and B were strikingly similar to data appearing in different form in other articles by different authors. Owing to the fact that the contentious data in the above article had already been published elsewhere, or were already under consideration for publication, prior to its submission to *Molecular Medicine Reports*, the Editor has decided that this paper should be retracted from the Journal. The authors were asked for an explanation to account for these concerns, but the Editorial Office did not receive any reply. The Editor apologizes to the readership for any inconvenience caused.

